# Cyclooxygenase-2: A Role in Cancer Stem Cell Survival and Repopulation of Cancer Cells during Therapy

**DOI:** 10.1155/2016/2048731

**Published:** 2016-11-01

**Authors:** Lisa Y. Pang, Emma A. Hurst, David J. Argyle

**Affiliations:** Royal (Dick) School of Veterinary Studies and Roslin Institute, The University of Edinburgh, Easter Bush, Midlothian EH25 9RG, UK

## Abstract

Cyclooxygenase-2 (COX-2) is an inducible form of the enzyme that catalyses the synthesis of prostanoids, including prostaglandin E2 (PGE_2_), a major mediator of inflammation and angiogenesis. COX-2 is overexpressed in cancer cells and is associated with progressive tumour growth, as well as resistance of cancer cells to conventional chemotherapy and radiotherapy. These therapies are often delivered in multiple doses, which are spaced out to allow the recovery of normal tissues between treatments. However, surviving cancer cells also proliferate during treatment intervals, leading to repopulation of the tumour and limiting the effectiveness of the treatment. Tumour cell repopulation is a major cause of treatment failure. The central dogma is that conventional chemotherapy and radiotherapy selects resistant cancer cells that are able to reinitiate tumour growth. However, there is compelling evidence of an active proliferative response, driven by increased COX-2 expression and downstream PGE_2_ release, which contribute to the repopulation of tumours and poor patient outcome. In this review, we will examine the evidence for a role of COX-2 in cancer stem cell biology and as a mediator of tumour repopulation that can be molecularly targeted to overcome resistance to therapy.

## 1. Introduction

To date, intensive cancer research has culminated in an increased knowledge of primary tumour formation, the development of sophisticated therapies, and prolonged survival time of cancer patients. However, cancer remains a common and lethal disease worldwide with tumour repopulation and metastasis as major causes of cancer-related deaths. A definitive cure for cancer patients will rely upon further molecular dissection and targeting of these two processes. In this regard, there is growing evidence that cancer is a stem cell disease, where tumours are composed of a mixture of genetically and functionally distinctive cells that contribute to tumour outgrowth, and a small population of cancer stem cells (CSCs) that can drive tumour initiation, therapy resistance, tumour repopulation, and metastasis. The CSC model posits that tumours are organised hierarchically in a similar, albeit distorted, manner as normal tissues. In a normal tissue, stem cells, at the apex of this hierarchy, give rise to transit amplifying cells, which proliferate rapidly and finally enter a postmitotic, differentiated state, in which the cells fulfill the various functions of the specific organ. CSCs share important properties with normal stem cells, including self-renewal and multilineage differentiation potential, and drive tumour progression as they have the exclusive ability to perpetuate indefinitely the growth of the tumour and give rise to a diverse array of differentiated progeny that make-up the bulk of the tumour mass [1–5]. Seminal work by Bonnet and Dick in 1997 first identified CSCs in acute myeloid leukemia [6], and subsequently CSCs have been isolated from a majority of solid malignancies including breast [5, 7–9], brain [10], colon [11], osteosarcoma [12, 13], squamous cell carcinoma [14, 15], and prostate [16, 17]. Most of these studies have defined CSCs functionally by their elevated tumour-initiating ability when inoculated into immune-deficient mice, relative to that of non-CSC cancer cells. Similarly to normal stem cells, CSCs are highly resistant to the cytotoxic effects of chemotherapy and radiotherapy and are able to reinitiate tumour growth [8, 9, 18]. This is seen clinically where these therapies do shrink the bulk of the tumour, but after a remission period of variable length, most patients do relapse with frequent development of drug resistance and metastatic dissemination.

Tumours are not only clonal outgrowths of deregulated cancer cells but potentiate their own progression and survival by fostering a complex and highly dynamic microenvironment, consisting of the extracellular matrix, endothelial cells, immune cells, and a plethora of cytokines and growth factors [19, 20]. Importantly, inflammatory cells and the cellular mediators of inflammation are prominent constituents of the microenvironment of all tumours [21]. In some cancers, the inflammatory conditions precede the development of malignancy, for example, inflammatory bowel disease is associated with colon cancer. Alternatively, an oncogenic change can drive tumour-promoting inflammation in tumours that are epidemiologically unrelated to overt inflammatory conditions [22, 23]. This “smoldering” inflammation in the microenvironment has many tumour-promoting effects including tissue remodelling, angiogenesis, cancer cell survival, metastasis, and immune evasion [21, 24]. One key inflammatory mediator deregulated in many cancers is cyclooxygenase-2 (COX-2). Elevated COX-2 expression, and that of its principle metabolic product prostaglandin E2 (PGE_2_), has been shown to be inversely associated with patient survival [25–27]. Epidemiological, clinical, and preclinical studies have shown that the inhibition of PGE_2_ synthesis through the use of either nonsteroidal anti-inflammatory drugs (NSAIDs) or specific COX-2 inhibitors has the potential to reduce the risk of developing certain cancers, including breast, head and neck squamous cell carcinoma, osteosarcoma, pancreas, and prostate cancers, and to reduce the mortality caused by these cancers [28–35]. Recently, PGE_2_ has been linked to the “phoenix rising pathway,” in which tissue damage initiates tissue repair [36]. In the context of common cancer therapies, which employ DNA damaging agents to trigger apoptosis, there is evidence that apoptotic cells release PGE_2_, a potent growth factor, that can stimulate the proliferation of surviving CSCs, leading to accelerated tumour repopulation and patient relapse [37, 38]. In this review, we will focus on the role of COX-2 in cancer stem cell biology, and as a mediator of tumour repopulation, and ultimately resistance to therapy.

## 2. COX-2 Plays a Central Role in Cancer

**Figure 1:**
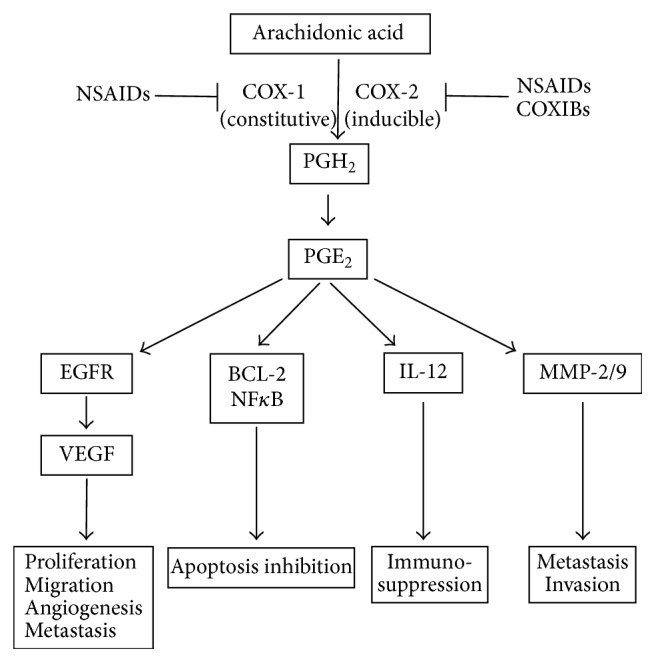
Prostaglandin E2 biosynthesis and downstream cellular effects. Arachidonic acid is released from cellular membranes and converted to PGH_2_ through the activity of the COX enzymes. COX-1 is constitutively expressed in many cells, generating low levels of prostaglandins that are cytoprotective and maintain homeostasis. In contrast, COX-2 is absent from most cells and is induced by a number of inflammatory stimuli. PGH_2 _is rapidly converted to PGE_2_, which plays a predominant role in cancer progression by stimulating tumour cell proliferation, migration, angiogenesis, apoptosis resistance, invasion, and metastasis. NSAIDS and COXIBS can pharmacologically block the activity of the COX enzymes.

Cyclooxygenases are enzymes necessary for the metabolic conversion of arachidonic acid to prostaglandins, including PGE_2_, a major mediator of inflammation and angiogenesis (Figure 1). PGE_2_ signals through four pharmacologically distinct G-protein coupled receptors, EP_1_, EP_2_, EP_3_, and EP_4_, which each activate different downstream signalling pathways. In turn, PGE_2_ is catabolized to the inactive 15-keto-PGE_2_ by the enzyme 15-hydroxyprostaglandin dehydrogenase [40, 41]. There are two isoforms of cyclooxygenase: COX-1 and COX-2. Both exist as integral, membrane-bound proteins, located primarily on the luminal side of the endoplasmic reticulum and nuclear envelope [42]. COX-1 is characterised as a housekeeping enzyme required for the maintenance of basal level prostaglandins [43] and is expressed constitutively in most tissues. It is responsible for the maintenance of internal homeostasis by participating in processes such as platelet aggregation, cytoprotection of the gastric mucosa, vascular smooth muscle functioning, and renal function. By contrast, COX-2 usually remains undetected in healthy tissues and organs. In adults, it is found only in the central nervous system, kidneys, vesicles, and placenta, whereas in the fetus, it occurs in the heart, kidneys, lungs, and skin [40, 44]. COX-2 is highly inducible and can be rapidly upregulated in response to various proinflammatory agents, including cytokines, mitogens, and tumour promoters, especially in cells involved in inflammation, pain, fever, Alzheimer's disease, osteoarthritis, or tumour formation [42, 45]. Under normal conditions, acute inflammation is a tightly controlled self-limiting response, where upon abatement of the inflammatory stimulus, specific cytokines, including interleukin-1 (IL-1) and IL-6, exert feedback inhibition causing COX-2 expression and PGE_2_ production to cease and the inflammatory response to subside. However, with sustained exposure to proinflammatory stimuli, continued expression of COX-2 leads to the transition from acute to chronic inflammation [42, 46]. In recent decades, COX-2 overexpression has been reported in several human cancers including breast [47–49], lung [47, 50], skin [51], colon [47, 52, 53], bone [32, 54, 55], cervical [56], oesophageal [57], pancreatic [58], prostate [59], and bladder cancer [60]. Constitutive expression of COX-2 and sustained biogenesis of PGE_2_ appear to play predominant roles in the initiation and promotion of cancer progression. PGE_2_ can mediate these effects through numerous signalling pathways including activation of vascular endothelial growth factor (VEGF) leading to increased cell proliferation, metastatic and invasive potential, and angiogenesis [61]; increased expression of the protooncogenes, BCL-2, and the epidermal growth factor receptor (EGFR), through the activation of the mitogen-activated protein kinase (MAPK) and the phosphoinositide 3-kinase (PI3K)/AKT pathway, respectively [62, 63]; increased transcriptional activity of the antiapoptotic mediator nuclear factor *κ*B (NF*κ*B) [64]; enhanced metastasis and invasion by activation of matrix metalloproteases (MMP-2 and MMP-9) [65]; and suppression of the production of IL-12, leading to immunosuppression [66].

Within the context of stem cell biology, PGE_2_ has been heralded as an evolutionarily conserved regulator of haematopoietic stem cells (HSCs) [67]. Stem cells are characterised by their unique abilities to both self-renew and differentiate to produce all mature cell lineages of a given tissue type [2]. In the adult vertebrate, HSCs reside in the bone marrow and are crucial to maintain lifelong production of all blood cells [68]. Utilising zebrafish and mouse models, the COX-2/PGE_2_ axis has been shown to be required for HSC formation [69], proliferation [70, 71], maintenance of the haematopoietic lineage [72], and bone marrow recovery following irradiation injury [69, 73]. Molecular dissection of the mechanisms by which PGE_2_ exerts these effects on HSCs has identified* in vivo* evidence that PGE_2_ enhances the activation of Wnt, a key regulator of stem cell self-renewal, during embryogenesis by stabilising *β*-catenin, and that Wnt-mediated regulation of HSC development is PGE_2_-dependent [67]. Given the stem-cell-enhancing activity of PGE_2_, and that PGE_2_ activates general cell survival and proliferation pathways, it is unsurprising that upregulation of COX-2 is associated with populations of CSCs isolated from several cancer types, including breast [74–77], colon [78, 79], and bone cancer [13, 80]. COX-2 is coexpressed with CSC markers including CD44, CD133, Oct3/4, LGR5, SOX-2, and ALDH [75, 78, 81–84]. A functional marker of CSCs is the ability to grow as spheroid colonies in defined serum-free culture conditions that supports the proliferation of undifferentiated cells [85]. Cells that overexpress COX-2 exhibit greater sphere forming efficiency and clonogenicity than those that express low levels of COX-2 [75, 86, 87]. In breast CSCs, isolated from the primary tumours of HER2/Neu transgenic mice, COX-2 expression was upregulated 30-fold in CSCs compared to non-CSCs, and constituted part of an eight-gene signature that correlated with breast cancer patient survival [88]. Furthermore, transfection of COX-2 into the ER-positive breast cancer cell line, MCF7, increased the ability of MCF7 cells to grow as spheres [86]. In our own work, we have shown that COX-2 expression is elevated 141-fold in the CSC population compared to the non-CSC population of canine osteosarcoma cells, and that COX-2 inhibition induced a dose-dependent decrease in sphere forming ability, indicating that COX-2 plays a major role in tumour initiation [13]. Our data is consistent with a previous study in which mouse embryonic stem cells lacking functional COX-2 have a normal growth rate and differentiation potential but are profoundly compromised in their ability to form teratocarcinomas* in vivo* [89]. Furthermore, CSCs isolated from human glioma cell lines, express constitutively high levels of COX-2 protein that correlates positively with radioresistance. Inhibition of COX-2 enhanced radiosensitivity of glioma CSCs and suppressed the expression of angiogenic and stemness-related genes [90]. Together, this data suggests that inhibiting COX-2 in CSCs reduces stem cell characteristics and that COX-2 plays a vital role in the maintenance and function of the CSC population.

## 3. Mechanisms of Resistance to Therapy

The death of all cancer cells in a tumour is the ultimate goal of cancer therapy. After surgery, radiotherapy and chemotherapy remain the most important treatment modalities of advanced carcinomas, and although they effectively shrink the tumour mass, some patients become progressively unresponsive and ultimately drug resistant [91]. Resistance can be divided into two broad groups: intrinsic or acquired. Intrinsic resistance indicates that, prior to receiving the therapy, resistance-mediating factors preexist in a subset of cancer cells that make the therapy ineffective, including increased drug efflux and aberrant DNA damage repair pathways. Acquired resistance can develop during treatment of tumours that were initially sensitive and can be caused by mutations arising during treatment, as well as through other adaptive responses, including activation of alternative compensatory signalling pathways and evasion of cell death [92]. Moreover, tumours contain a high degree of molecular heterogeneity; thus, drug resistance can arise through therapy-induced selection of a resistant population of cells that was present in the original tumour [2]. Recent studies have shown that the extensive heterogeneity observed within tumours occurs through mechanisms independent of CSC differentiation and supports a model whereby the CSC phenotype is dynamic rather than a fixed state. For example, induction of epithelial-to-mesenchymal transition (EMT), by ectopic expression of the transcription factors Twist or Snail, in mammary cancer cells is associated with CSC qualities and an increased propensity to form tumours [93, 94]. Similarly, melanoma cells can reversibly turn on and off the histone demethylase JARID1B, and cells that express JARID1B are more tumourigenic than those that do not [95]. And exposure of glioma cells to perivascular nitric oxide reversibly promotes their ability to form tumours [96]. These findings challenge the unidirectional hierarchical CSC model: signifying that non-CSCs can dedifferentiate and can acquire CSC-like properties under certain conditions.

Common cancer therapies produce toxic substances that destroy crucial cellular macromolecules, including DNA, leading to cell death. An unfortunate side effect is high toxicity to normal tissues: to avoid severe toxic reactions, radiotherapy and chemotherapy are often given in multiple doses, which are spaced out to allow the repopulation of surviving cells in normal tissues during the prolonged overall treatment time. However, surviving cancer cells also proliferate during the intervals between treatments and this process of repopulation is an important cause of treatment failure [97]. Furthermore, a long recognised phenomenon is that of accelerated repopulation, where the few surviving cancer cells that have escaped death after exposure to radiotherapy or chemotherapy can rapidly repopulate the badly damaged tumour by proliferating at a markedly accelerated rate. Subsequent tumour repopulation with resistant cancer cells often results in a more aggressive cancer phenotype with poor prognosis for the patient. There is ambiguity regarding tumour type and repopulation potential: some studies report that accelerated repopulation occurs only in the late stages of radiation treatment, whereas other studies, such as those in cervical cancer [98], squamous cell carcinomas [99], bladder cancer [100], and colorectal carcinomas [101], show that the onset time of accelerated repopulation is relatively short [102, 103]. This has implications for treatment regimes, and the efficacy of therapy. The molecular mechanisms underlying this process of accelerated tumour repopulation are not well understood. A seminal study has shown that CSCs, isolated from bladder urothelial carcinomas, actively proliferate in response to chemotherapy-induced damages and repopulate residual tumours between chemotherapy cycles, in a similar fashion to how normal tissue stem cells mobilise to the site of a wound during tissue repair [37].

Wound healing and tissue regeneration are essential processes for all multicellular organisms. Some organisms have the ability to entirely regenerate amputated limbs, such as salamanders [104], whereas other organisms can only partially replace damaged organs, such as humans [105]. The resident tissue stem cells play a crucial role in wound healing and tissue regeneration. Damaged tissues mobilise and recruit stem cells to the site of damage, where they proliferate, differentiate, and eventually replace the damaged tissue [106]. Several studies in* Drosophila *[107],* Xenopus *[108],* Planaria* [109], and* Hydra* [110] have indicated a facilitative role for apoptosis as a trigger for tissue remodelling in response to tissue injury, identifying caspase activation as a key requirement for cell proliferation and stem cell recruitment. Although counterintuitive, as apoptosis is generally considered as a means for multicellular organisms to dispose of damaged or unwanted cells, there is increasing evidence that dying cells can signal their presence to the surrounding tissues and, in doing so, elicit tissue repair and regeneration that compensates for any loss of function caused by cell death [111]. The first evidence of this in a mammalian model was provided by Li et al. [36] and coined the phrase “phoenix rising pathway,” in which tissue damage initiates tissue repair. This study revealed that mice deficient in caspase-3 and caspase-7, which are essential apoptotic proteases, exhibited reduced rates of tissue repair in dorsal skin wounds and defects in liver regeneration following partial hepatectomy. Mechanistically, apoptotic cells released PGE_2_ in a caspase-dependent manner, and this in turn stimulates stem cell proliferation and tissue regeneration [36]. Given that aberrant apoptosis is a hallmark of cancer [4] and that activation of caspases to induce apoptosis is the prevailing ideology of most cancer treatments, what is the role of apoptosis-induced compensatory proliferation in cancer development? Is the phoenix rising pathway clinically relevant?

**Figure 2:**
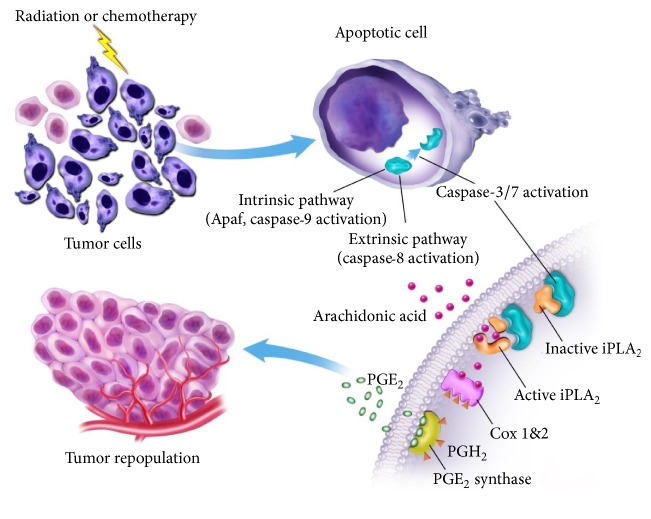
Schematic representation of apoptosis-mediated tumour cell repopulation. In tumours damaged by cytotoxic therapies apoptotic cells activate caspase-3 and caspase-7 through either intrinsic pathways, involving Apaf and caspase-9 activation, or extrinsic pathways, involving caspase-8 activation. Activated caspase-3 and caspase-7 activate calcium-independent phospholipase A2 (iPLA_2_), which increases the synthesis and release of arachidonic acid. Arachidonic acid is then converted into PGH_2_ by COX-1 and COX-2, which is subsequently converted into PGE_2_ by the enzyme PGE_2_ synthase. PGE_2_ stimulated cancer stem cell proliferation and tumour repopulation (figure was adapted from [39] with the permission of Professor Li).

Cancers often acquire mutations that prevent apoptosis, leading to the survival of precancerous cells (that would otherwise die) and giving rise to neoplasia. This model appears to be oversimplified, and data is emerging, which expands and links the role of apoptosis-induced compensatory proliferation in normal tissue repair and regeneration, to tumourigenesis. Studies of *γ*-irradiation-induced lymphoma formation in mice deficient in PUMA, a DNA damage induced proapoptotic mediator, showed a reduction in apoptosis and, surprisingly, a concurrent reduction in tumour incidence [112, 113]. Similarly, PUMA-deficient mice treated with diethylnitrosamine, a DNA-alkylating agent and hepatocarcinogen, showed a reduction in apoptosis of hepatocytes and decreased tumour incidence [114]. Although these studies lack mechanistic insight, this data indicates that PUMA-dependent apoptotic cell death may drive compensatory proliferation in lymphogenesis and hepatocarcinogenesis. Further to this, a seminal study by Huang et al. [39] illustrated,* in vitro* and* in vivo*, that the phoenix rising pathway is applicable to cancer biology, whereby apoptotic tumour cells can stimulate the repopulation of tumours from a small number of surviving cells, and that this process is caspase-3-dependent and involves upregulation of arachidonic acid and subsequent PGE_2_ production (Figure 2). In this study, labelled cancer cells were implanted into mice with or without lethally irradiated, apoptotic, mouse embryonic fibroblast cells (MEFs). The presence of apoptotic cells increased cell proliferation and tumour cell growth. However implantation of irradiated, caspase-3-deficient MEFs ablated these results. Exogenous treatment with PGE_2_ also caused cancer cells to grow at a faster rate than untreated cells. Subsequently, Allen et al. [115] utilised a panel of established human cancer cell lines to show that IR-induced, caspase-3-dependent, PGE_2_ production is a common response of irradiated tumour cells and that PGE_2_ production generally correlated with enhanced growth of cells that survive irradiation and of unirradiated cells cocultured with irradiated cells. Therefore, the caspase-3/PGE_2_ axis is a direct link between cell death and tumour repopulation, highlighting that tumours may exploit a tissue homeostatic mechanism to preserve themselves when damaged by cytotoxic therapy, and given the high radiation doses required to kill high numbers of tumour cells, the rare surviving cells are likely to experience significant DNA damage, and rapid proliferation of such cells may enhance mutagenesis and drive tumour progression toward a more metastatic state.

## 4. Implications for Cancer Management and Therapy

There is accumulating evidence that the phoenix rising pathway is clinically relevant. In patients with head and neck cancer, high amounts of activated caspase-3 were correlated with high rates of tumour recurrence, and in patients with breast cancer, high caspase-3 levels were correlated with shorter survival time [39]. This has implications for cancer therapy: efforts to develop agents that activate caspases must be reexamined; and small molecule inhibitors of caspases should now be evaluated for their properties to enhance cancer chemotherapy or radiotherapy. Human clinical trials are currently lacking in this area, but several studies using mouse models indicate that blocking apoptosis may increase sensitivity to radiotherapy. For example, in a mouse model of lung cancer, treatment with a caspase-3 inhibitor led to an increase in autophagy and radiosensitivity [116]; and treatment with a pan-caspase inhibitor, zVAD, or small interfering RNA directed against caspase-3 and caspase-7 led to radiosensitivity and delayed tumour growth rates, in breast and lung xenografts [117]. In addition, a recent study has shown that low doses of radiation cause partial caspase-3 activation that leads to genome instability, both* in vitro* and* in vivo*, through the generation of persistent DNA strand breaks [118]. These findings implicate caspase-3 as a promising target to improve chemo- and radiation-therapy outcomes. However, inhibition of caspase-dependent apoptosis is counterintuitive, when taking into consideration that the aim of cytotoxic therapies is to induce apoptosis of cancer cells, and further research is required to determine if, in the absence of caspase-activity, cell death comes by an alternative pathway, such as necrotic cell death or senescence.

An alternative strategy is to prevent compensatory proliferation by targeting PGE_2_ production, which is downstream from caspase-3, by chemical inhibition of COX-1 and COX-2. NSAIDS effectively target cyclooxygenase enzymes and are widely used to treat common inflammatory diseases; for example, naproxen and ibuprofen are widely used and well tolerated. Selective COX-2 inhibitors, such as celecoxib, have been marketed but many have been withdrawn due to increased risk of myocardial infarction; however, the risk has not been assessed for short-term courses during cancer therapy. As we have previously discussed, overexpression of COX-2 and chronic inflammation has been attributed to the development of several cancer types, and there has been extensive preclinical and epidemiological studies that support the targeting of the COX-2 pathway for the prevention and treatment of malignancy (reviewed extensively here [119–121]). Several mouse studies have provided evidence of the synergistic effect of COX-2 inhibitors in combination with chemotherapy and radiotherapy including mouse models of nasopharyngeal carcinoma [122], pancreatic adenocarcinoma [123], and medulloblastoma-derived CSCs [124]. In the context of tumour repopulation, a seminal study by Kurtova et al. [37] used a human bladder cancer xenograft model to show that there is selective repopulation of the tumour from previously slowly proliferating bladder tumour cells that have markers of CSCs. This is the first study to effectively show that CSCs actively contribute to therapeutic resistance via the phoenix rising pathway, whereby chemotherapy effectively induces apoptosis and associated PGE_2_ release then promotes neighbouring CSC repopulation, akin to how tissue resident stem cells mobilise to wound sites during tissue repair. Additional studies have supported that repopulation can be abrogated by COX-2 inhibition of PGE_2_-signalling: treatment of a panel of human cancer cell lines with the pan COX-1 and COX-2 inhibitor, indomethacin, blocked radiation-induced PGE_2_ production, and inhibited cancer cell proliferation [115]; a study of prostate adenocarcinoma mouse xenografts showed that topical application of the NSAID, diclofenac, significantly reduced tumour growth in combination with 3 Gy irradiation [125]; and significantly, celecoxib delivered between rounds of gemcitabine and cisplatin substantially suppressed bladder urothelial carcinoma xenograft regrowth and enhanced the chemotherapeutic response in xenografts from a chemoresistant patient [37]. These results advocate that PGE_2_-stimulated tumour repopulation is a critical issue to consider during treatment planning and that prospective clinical trials are needed to define the effect of COX-2 inhibition on tumour repopulation between cycles of chemotherapy or radiotherapy. Several clinical studies have been conducted in which celecoxib has been used in combination with standard chemotherapy and radiotherapy and show varying results depending on the type and stage of cancer [119]. A meta-analysis of these trials showed a modest activity against advanced cancers, but no significant effect on one-year survival rate [126]. These studies were not in the context of blocking apoptosis-induced compensatory proliferation to enhance the efficacy of therapy. Future studies to address this clinical problem are needed to define optimum treatment regimes, the sensitivity of the CSC population to specific COX-2 inhibitors, and to determine different pharmacokinetics and pharmacodynamics associated with chemotherapy, as some drugs may be better or poorer induces of tumour repopulation. There is also a great diversity of tumours, and it is likely that some tumour types may be more reciprocal to apoptosis-driven tumour repopulation than others.

NSAIDS and selective COX-2 inhibitors target not only COX2 activity but also suppress the biosynthesis of other physiologically important prostanoids that are associated with the adverse effects of these drugs on cardiovascular function including increased risk of myocardial infraction and stroke [127]; therefore, alternative strategies to inhibit PGE_2_ production that would negate the cardiovascular risk and lead to safer therapeutic tools are currently being investigated, including selective targeting of individual prostanoids via inhibition of their corresponding terminal synthases and disruption of platelet-driven COX2 induction.

An attractive target for regulation of PGE_2_ levels is inhibition of microsomal prostaglandin E synthase-1 (mPGES-1). mPGES-1 is the inducible terminal synthase in PGE_2_ biosynthesis and is functionally coupled to COX-2: the induction of these two enzymes leads to increased PGE_2_ production [128]. In cancer, mPGES-1 is overexpressed in a number of cancers including gastrointestinal, lung, stomach, brain, breast, pancreas, prostate, and papillary thyroid carcinoma [129]. Moreover, mPGES-1 expression is associated with vascular invasion and poor prognosis in colorectal cancer [130]. The therapeutic potential of mPGES-1 has been proved in multiple studies using mice models with genetic deletion of mPGES-1, in which there was a decrease in the production of PGE_2_ and associated pain, fever, inflammation, and tumourigenesis [131–133]. Currently there are no selective mPGES-1 inhibitors available for clinical use. However, research is ongoing to identify and characterise specific mPGES-1 inhibitors, and we would advocate that this research should extend to studies looking at these drugs in combination radiotherapy and the impact on apoptosis-driven tumour cell repopulation.

Similarly, a growing body of evidence supports the central role of platelets in metastasis. There is extensive cross-talk between platelets and cancer cells, whereby tumours can stimulate platelet activation and activated platelets, in turn, promote tumour growth and metastasis. A central event involves an aberrant expression of COX-2 in the cancer cells, which influences cell-cycle progression and contributes to the acquisition of a cell migratory phenotype through the induction of EMT gene expression profile. Platelets are also activated in response to wound healing, and they secrete a number of factors that are important mediators of tissue remodelling at the site of injury. By extension, future research should focus on the role of platelets in the tumour microenvironment, the effect on the phoenix rising pathway and to determine if pharmacological inhibition of platelet function could prevent tumour repopulation and metastasis.

## 5. Conclusions

Recent evidence has challenged the paradigm that apoptosis is a barrier for carcinogenesis: the common goal of standard chemotherapy and radiotherapy is to kill cancer cells by inducing apoptosis. However, apoptosis may be a double-edged sword, leading initially to increased cell death of cancer cells, but accompanied by increased PGE_2_ secretion and subsequent growth stimulation of CSCs and repopulation of the tumour. Repopulation during chemotherapy and radiotherapy has long been recognised as an important cause of treatment failure. Here we have presented the phoenix rising pathway as a potential driver of compensatory proliferation, and advocated that the ability of COX-2 inhibitors to selectively inhibit the proliferation of tumour cells during therapy should be evaluated. Further understanding of the biological processes of compensatory cell proliferation may give therapeutically beneficial insight into tissue repair and aid the development of new strategies to inhibit tumour repopulation during therapy and improve clinical outcomes.
